# Heterotrimeric G protein signalling in the plant kingdom

**DOI:** 10.1098/rsob.120186

**Published:** 2013-03

**Authors:** Daisuke Urano, Jin-Gui Chen, José Ramón Botella, Alan M. Jones

**Affiliations:** 1 Department of Biology, University of North Carolina, Chapel Hill, NC 27599, USA; 2 Department of Pharmacology, University of North Carolina, Chapel Hill, NC 27599, USA; 3 Biosciences Division, Oak Ridge National Laboratory, Oak Ridge, TN 37831, USA; 4 Plant Genetic Engineering Laboratory, School of Agriculture and Food Sciences, University of Queensland, Brisbane, Queensland 4072, Australia

**Keywords:** heterotrimeric G protein, plant, review

## Abstract

In animals, heterotrimeric G proteins, comprising α-, β-and γ-subunits, perceive extracellular stimuli through cell surface receptors, and transmit signals to ion channels, enzymes and other effector proteins to affect numerous cellular behaviours. In plants, G proteins have structural similarities to the corresponding molecules in animals but transmit signals by atypical mechanisms and effector proteins to control growth, cell proliferation, defence, stomate movements, channel regulation, sugar sensing and some hormonal responses. In this review, we summarize the current knowledge on the molecular regulation of plant G proteins, their effectors and the physiological functions studied mainly in two model organisms: *Arabidopsis thaliana* and rice (*Oryza sativa*). We also look at recent progress on structural analyses, systems biology and evolutionary studies.

## 2. Introduction: history of G protein research in plants

**Figure 1.**
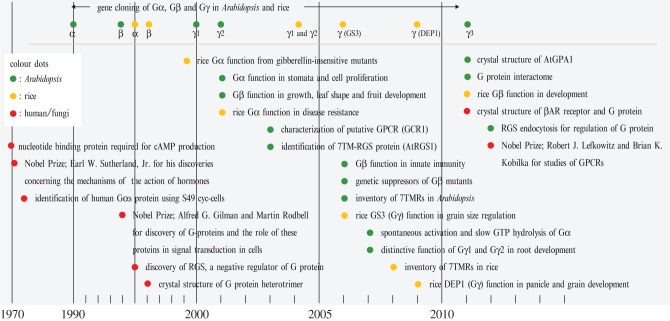
History of plant G protein science. In the 1970s, G proteins were identified as a signal transducer connecting the hormone receptor and the adenylyl cyclase in mammals. In the early 1990s, plant G protein genes were cloned and shown to have conserved domains and motifs with the animal genes. In the late 1990s, much effort went towards physiological roles of G proteins using genetics. In the 2000s, the Gβγ-subunits, the regulators (GPCR-like genes and a 7TM-RGS gene) and effectors of G protein were cloned and characterized genetically and biochemically. In 2007, the ‘self-activating’ property of the plant G protein was revealed. In addition, the transcriptome, proteome and interactome analyses revealed comprehensive knowledge of the plant G protein pathways. In the last few years, the crystal structure and computational simulation solved the mechanism of self-activation. Publications on the physiological functions and signalling components of G protein pathways are exponentially increasing, providing evidence for their important and divergent functions in plants.

In animals, heterotrimeric G proteins transmit extracellular signals, such as hormones, neurotransmitters, chemokines, lipid mediators, light, tastes and odorants, into intracellular signalling components [1,2]. In the early 1970s, Martin Rodbell, a Nobel Prize winner in 1994, suggested three biological machines—discriminator, transducer and amplifier—needed to produce cAMP after cells perceive the hormone glucagon [3] (figure 1). This novel concept of its time materialized from Rodbell's experience as a Navy radioman [3]. With exquisite biochemistries, the discriminator and the amplifier became the seven transmembrane G-protein-coupled receptor (GPCR) and adenylyl cyclase [1], respectively. The signal transducer became the heterotrimeric G protein connecting the receptor to the amplifier by another Nobel Prize winner, Alfred G. Gilman [4], and his colleagues (see also Lefkowitz [5] for a historical review of G protein research). The GPCRs were discovered and characterized as membrane-localized hormone receptors, using radio-labelled ligands in the 1960s and 1970s [5]. The crystal structures revealed the detailed action of how GPCRs receive hormones and activate the heterotrimeric G protein [6]. For these studies on GPCRs, the 2012 Nobel Prize in chemistry was awarded to two more G protein scientists: Robert J. Lefkowitz and Brian K. Kobilka.

In plants, the first α-subunit of G protein was cloned from *Arabidopsis* (AtGPA1) in 1990 [7] and later from other species [8–12]. The physiological roles were determined using loss-of-function mutants and transgenic lines in stomatal opening/closure [13–15], fungal defence [16–18], oxidative stress [19,20], seed germination [21,22], sugar perception [21,23], some phytochrome/cryptochrome-mediated responses [24] (but not all [25]), and seedling and root development [24,26,27]. In rice, the Gα-subunit (RGA1)-deficient line, named *dwarf1* (*d1*) mutant, was found in a screen for mutants defective in gibberellin (GA) responsiveness [28,29]. Subsequently, Gβ and Gγ genes were cloned in *Arabidopsis* [30–32], rice [33,34] and others [11,30,35]. A general physiological function for Gβ was proposed from the phenotype of an *Arabidopsis* mutant having an altered development of leaves, flowers and fruits [36]. We now know that Gβ functions expand to the root [37], ion channels, stoma [38] and fungal defence [18,39]. Gβ- and Gγ-deficient mutants share some developmental phenotypes (e.g. rounded leaf shape and sugar sensitivity) with Gα mutants, but also differ in others (e.g. lateral root production and fungal defence) [36,37]. In animals, phenotypes shared by both Gα and Gβ mutations are indicated to be disruptions in pathways in which the predominant transducer is the Gα-subunit. Opposite phenotypes reveal phenotypes in which the Gβγ dimer is the predominant transducer. Thus, plant G protein research initially tried to extrapolate from the vast amount of knowledge accumulated in animal systems.

However, findings from two different directions came to light by the mid-2000s, indicating that plant G proteins use a regulatory system distinct from animal G proteins [23,40] (see §3). In 2003, the GTPase-accelerating protein (GAP) of regulator of G protein signalling (RGS) protein, AtRGS1, stood out for its hybrid topology [23]. AtRGS1 contains seven N-terminal transmembrane helices (7TM) like a GPCR and a C-terminal RGS box typically found in cytoplasmic animal RGS proteins [23,40]. Such a chimaera between a GPCR and RGS protein had never been reported before; the animal G protein field looked over *Arabidopsis* G signalling with great curiosity and puzzlement.

The second clue that plant G protein signalling is different from the animal paradigm came in 2007 when Francis Willard and co-workers showed that the *Arabidopsis* Gα-subunit spontaneously bound GTP *in vitro*; a guanine nucleotide exchange factor (GEF) was not needed for activation [40]. This ‘self-activating’ property, a term coined in subsequent publications and described in greater detail below, suggests that plant G proteins do not need, and therefore do not have, GPCRs; a blasphemous notion in the G protein field. Nonetheless, biochemical, structural, evolutionary and computational analyses leave no other conclusion: the plant kingdom uses a distinct regulatory system in G signalling [41–43].

## 3. Regulatory system of animal and plant heterotrimeric G proteins

### 3.1. Basic G protein concept based on its biochemical activity

**Figure 2.**
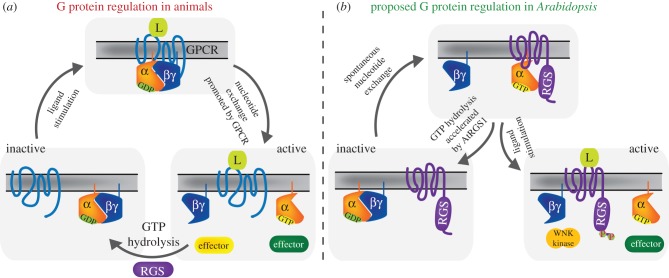
The ‘G’ cycle of animals versus *Arabidopsis*. (*a*) G protein regulation in mammalian cells. In the absence of ligand, G protein forms an inactive heterotrimer with Gβγ dimer (bottom left). Ligand-bound GPCR promotes GDP dissociation and GTP binding on G protein (top). GTP-bound Gα dissociates from Gβγ dimer, and both activated Gα and freely released Gβγ modulate activity of the effectors (bottom right). Gα hydrolyses GTP to GDP, and re-binds to Gβγ to return to its inactive state. (*b*) G protein regulation modelled in *Arabidopsis. Arabidopsis* G protein (AtGPA1) can spontaneously dissociate GDP and activate itself (bottom left). AtGPA1 does not hydrolyse its GDP rapidly; however, AtRGS1, a 7TM-RGS protein, promotes the GTP hydrolysis of AtGPA1 (top). d-glucose or other stimuli functions on AtRGS1 directly or indirectly, and decouples AtGPA1 from AtRGS1 (bottom right). Once released from AtRGS1, AtGPA1 does not hydrolyse its GTP efficiently, maintaining its active state and modulating the effector activities.

Figure 2 compares the regulatory system of heterotrimeric G proteins in animals versus most plants. In mammals, the cognate heterotrimeric G protein is activated by a GPCR or other GEF [1]. At steady state, the α-subunit of G protein keeps its GDP tightly bound and forms an inactive heterotrimer with the Gβγ-subunits (figure 2*a*, bottom left) [1]. An agonist-stimulated GPCR promotes GDP dissociation from the α-subunit (figure 2*a*, top), and the nucleotide-free Gα interacts with GTP, which has a concentration 10 times higher than that of GDP in animal cells. This is a rate-limiting step in the animal G protein cycle. The newly GTP-bound Gα changes its conformation to the active form, consequently dissociating from Gβγ, and interacts with and regulates the activity of its effectors (figure 2*a*, bottom right). Known animal effectors are adenylyl cyclases, phospholipase Cβ and RGS-RhoGEFs [2], and there are many more (see fig. 1 of [44]). The active Gα-subunit returns to the inactive state by hydrolysing the bound GTP. In this reaction, RGS proteins or other GAPs promote the GTP hydrolysis and terminate G protein signalling. Gα mutants with enhanced activity of GDP dissociation [45] or abolished GTPase activity [46] function as constitutively active G proteins. This suggests that both slow GDP dissociation and rapid GTP hydrolysis are required for keeping the heterotrimer inactive and for the proper signal transduction in animal cells. The activity of RGS proteins also increases the initial amplitude of G signalling by a process called dynamic scaffolding [47].

Although plant G protein signalling uses similar elements, the cycle starkly contrasts with the animal model. In plants, the α-subunit of the G protein spontaneously releases GDP (figure 2*b*, arrow from bottom left to top) and forms a stable GTP-bound state [40,41,48]. The exchange rate of GDP for GTP in AtGPA1 (*k*_on_ = 1.4–14.4 min^−1^ [40,41,48]) is comparable with a constitutively active mutant of human Gαs (Gαs S366A, *k*_on_ = 14 min^−1^) [45]. Moreover, the intrinsic GTPase activity of the plant AtGPA1 (*k*_cat_ = 0.03–0.12 min^−1^ [40,41,48]) is much lower than mammals (human Gαs, *k*_cat_ = 3.5 min^−1^ [49]), being close to the GTPase-crippled mutant Gαs Q227L (the *k*_cat_ is probably approx. 0.06 min^−1^ [46]). While the original rate constants were determined using rice and *Arabidopsis* G proteins, we recently showed that this self-activating property is found throughout the plant kingdom [48]. In *Arabidopsis*, a 7TM-RGS protein, AtRGS1 promotes GTP hydrolysis of the α-subunit [40,50], resulting in the formation of an inactive heterotrimer (figure 2*b*, top). Genetic evidence is consistent with d-glucose being the ligand that halts AtRGS1 GAP activity and, by doing so, allowing AtGPA1 to self-activate (figure 2*b*, bottom right) [23,40,51,52]. The detailed mechanism for this atypical activation mechanism [53] is described in greater detail in §3.2. Back to the bigger picture, in animals, different ligands stimulate the stimulator (GPCR), whereas it appears that in plants the ligands inhibit the inhibitor (e.g. AtRGS1 in *Arabidopsis*). ‘Inhibiting the inhibitor’ seems to be a common theme for receptor regulation in plants [54].

### 3.2. Endocytosis of 7TM receptors in mammals and plants: same actions, different reactions

**Figure 3.**
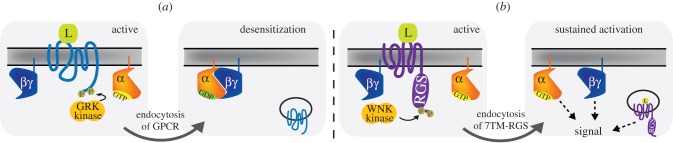
Endocytosis of 7TMR in animals versus *Arabidopsis*. (*a*) In animals, ligand-stimulated GPCRs are phosphorylated by G protein-receptor kinases (GRKs) or other kinases. The phosphorylated receptors are recognized by β-arrestin, and then endocytosed by a clathrin complex. The endocytosed receptors are not able to perceive extracellular ligands. Cells are thereby desensitized. (*b*) In *Arabidopsis*, 7TM-RGS is phosphorylated at the carboxyl-terminus by WNK-family kinases. Phosphorylation triggers endocytosis of 7TM-RGS. The endocytosis of 7TM-RGS is probably used for sustained activation of G protein signalling on plasma membrane and for a G-protein-independent pathway from endosomal RGS protein.

Many types of cell possess feedback systems to fine-tune the strength, duration and specificity of signals. In mammals, GPCRs are internalized to desensitize in response to excessive and/or continuous stimuli (figure 3*a*) [5]. Such a mechanism is important to protect cells from harmful doses of the ligands. Some GPCRs are phosphorylated at the carboxyl-terminal region by kinases, such as G-protein receptor kinases (GRKs; figure 3). The phosphorylated GPCRs are recognized by β-arrestin, which functions as an adaptor connecting GPCRs to the endocytic machinery. Then, GPCRs are endocytosed by clathrin-dependent and adaptor protein 2 (AP2)-complex-dependent mechanisms.

Similar to mammalian GPCRs, the plant 7TM-RGS1 is trafficked rapidly from the plasma membrane to the endosome upon d-glucose or other sugar treatments (figure 3*b*) [53,55]. Like mammalian GPCRs, AtRGS1 is phosphorylated at the C-terminus, shown to be essential for AtRGS1 endocytosis. Although plant genomes do not encode GRK homologues, a WITH NO LYSINE kinase (WNK), AtWNK8, found among the G protein interactome (discussed below), phosphorylates AtRGS1 for endocytosis. Because clathrin-dependent and AP2-complex-dependent systems are conserved between mammals and plants, these trafficking components are possibly critical for AtRGS1 endocytosis. AtGPA1 remains on the plasma membrane, thus AtRGS1 and AtGPA1 become physically uncoupled allowing AtGPA1 to self-activate (figure 3) [53]. Because loss-of-function mutations in AtRGS1 do not confer constitutive sugar signalling, the story is more complex. One explanation is that sugar signalling through activated AtGPA1 at the plasma membrane also requires an origin of signalling through AtRGS1 at the endosome [53].

### 3.3. Structure and mechanism of plant ‘self-activating’ G protein

**Figure 4.**
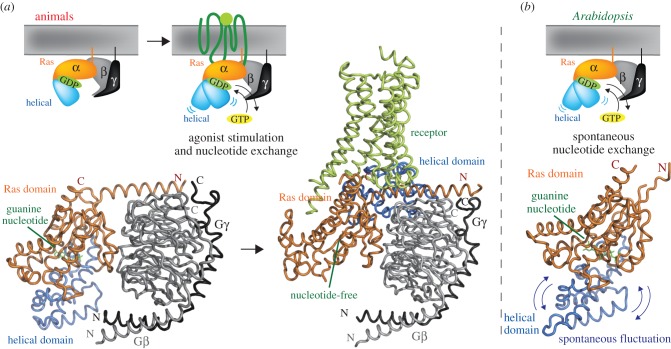
Crystal structure and activation mechanisms of G protein. (*a*) Structural basis of animal G protein activation. Left: Gα protein forms stable heterotrimer with Gβγ dimer (grey and black) at the steady state. GDP (green) is tightly bound to a Ras domain (orange) of the α-subunit, and covered by the helical domain (sky blue). Right: in the presence of ligand-bound receptor, the helical domain moves and changes orientation. The structural change causes GDP dissociation from the α-subunit, the subsequent GTP binding and activation. (*b*) Structure of *Arabidopsis* AtGPA1 is entirely similar to mammalian Gα proteins. However, the helical domain of AtGPA1 fluctuates spontaneously. The spontaneous fluctuation initiates GDP dissociation, and nucleotide exchange. Crystal structures shown are animal heterotrimeric G protein (PDB: 1GOT) [56], G protein and β2 adrenergic receptor (PDB: 3SN6) [6], and *Arabidopsis* AtGPA1 (PDB: 2XTZ) [41]. The cartoon for the animal model was adapted from Rasmussen *et al*. [6].

**Figure 5.**
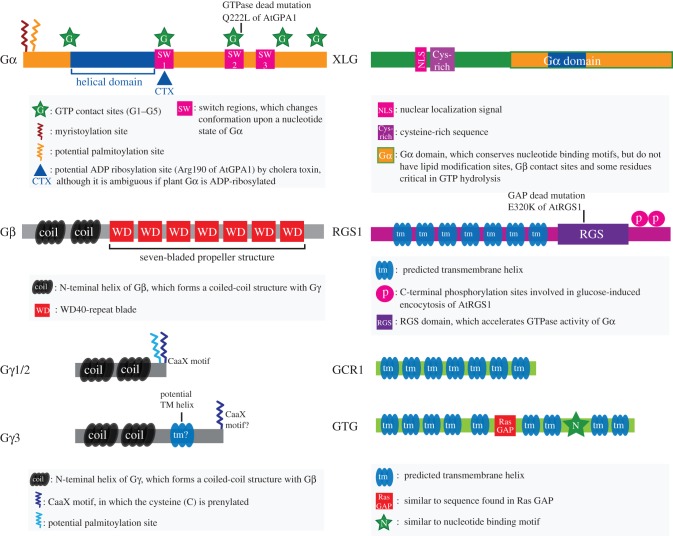
Domain structures of *Arabidopsis* G protein-related proteins. AtGPA1, a canonical Gα-subunit, is composed of a Ras-homology domain and a helical domain. Gα sequence contains N-terminal lipid modification sites, three switch regions and guanine nucleotide binding motifs. Gα has a conserved asparagine for cholera toxin (CTX), although there has been no evidence that CTX ADP-ribosylates plant Gα-subunits. AGB1, a Gβ-subunit, harbours N-terminal coiled-coil helices and a WD40 repeat propeller. Typical (AGG1 or AGG2) and atypical (AGG3) Gγ-subunits: typical Gγ-subunit has a coiled-coil region to form a dimer with Gβ and a C-terminal CaaX motif for a lipid modification; atypical Gγ3 has a potential transmembrane helix in the middle, a cysteine-rich sequence in the C-terminal region and a putative CaaX motif. Notably, the CaaX motif of AGG3 is not conserved in some other plants. XLG, a plant-specific Gα-like protein, has a nuclear localization signal (NLS), cysteine-rich region and a C-terminal Gα-like domain [57]. The Gα-like sequence does not conserve some of the residues for hydrolysing GTP or for interacting with Gβγ [57,58]. AtRGS1 is a 7TM protein harbouring a RGS domain; the 7TM region is essential for localizing RGS1 to the plasma membrane [52]. The RGS domain binds to the Gα-subunit and accelerates the GTPase activity [23]. The C-terminal phosphorylation sites are critical in its endocytosis [53]. AtGCR1 is a 7TM protein similar to a slime mould cAMP receptor; the C-terminal region was essential in the Gα interaction [59]. GTG is a GPCR-type GTP-binding protein; AtGTG1 and AtGTG2 possess nine potential transmembrane helices, a homologous region to mammalian RasGAP protein and a nucleotide binding motif-like sequence [60]. The human homologue functions as a pH-dependent anion channel [61], but the structural basis has not been analysed.

Figures 4 and 5 show crystal structures and domain architectures of the G protein heterotrimer and the related proteins. The α-subunit of the G protein complex is composed of distinctive helical and Ras domains (see *Arabidopsis* AtGPA1 structure in figure 4*b*). The Ras domain contains motifs to bind guanine nucleotide, Gβγ dimer [62], GPCRs and effectors, whereas the helical domain shelters the guanine nucleotide binding pocket. The β-subunit contains an amino-terminal coiled-coil motif and a carboxyl-terminal WD40 repeat domain [62] (figures 4*a* and 5). The amino-terminus of Gβ forms the stable coiled-coil interaction with the γ-subunit [62], and the WD40 repeat domain contains the effector and Gα binding surface (figure 4*a*). The effector binding surface on Gβ is normally masked by the GDP-bound form of Gα protein; therefore, Gβγ is active only after dissociating from the α-subunit [2]. The γ-subunit is a small (normally less than 100 residue) protein containing a coiled-coil region and a prenylation site at its carboxyl-terminus [1] (figures 4*a* and 5), which is required for its membrane targeting [63,64]. This is described in greater detail below.

The human Gα structure is remarkedly altered, depending on the presence or absence of a GPCR [6]. As described earlier, the helical domain of the Gα-subunit covers the guanine nucleotide (figure 4*a*, left), however, the helical domain is oriented by agonist-bound GPCR (figure 4*a*, right), and the structural change discloses the guanine nucleotide binding pocket on the Ras domain and promotes GDP dissociation from the Gα protein [6]. *Arabidopsis* G protein rapidly dissociates its GDP without GEF proteins [35]. As shown in figure 4*b*, the crystal structure of AtGPA1 is essentially the same as the human inhibitory Gα protein, Gαi1 (the root-mean-squared deviation is only 1.8 Å) [36], but the helical domain of AtGPA1 is the structure that imparts the rapid GDP dissociation property [36]. Molecular dynamics simulations of the AtGPA1 structure indicate spontaneous fluctuation of the *Arabidopsis* helical domain in the absence of a GPCR [38], suggesting the conserved role of the helical domain to determine nucleotide dissociation from the α-subunit. Overall, the plant G protein rapidly exchanges its guanine nucleotide owing to the spontaneous fluctuation of the helical domain that is normally promoted by agonist-bound GPCR in animals.

### 3.4. G-protein-coupled receptor candidates proposed in the plant kingdom

In the race to find plant GPCRs, there were many stumbles. Several GPCR-type candidates were initially proposed but later discredited. GCR1 [11,59,65–67], GCR2 [68], mildew resistance locus O (MLO) proteins [69,70], GPCR-type G protein (GTG) 1 and GTG2 [60], and some others [71,72] were proposed to be GPCRs based on the predicted or proved membrane topology, and some G-protein-related phenotypes were genetically shown in *Arabidopsis* providing some support. GCR1 shares homology with the cAMP receptor (cAR1) of *Dictyostelium discoideum* [65] and was reported to interact with AtGPA1 [59]. Both G protein genes and GCR1 are involved in abscisic acid (ABA)-dependent stomate closure (see §5 for details) [13,59]. However, direct GEF activity of GCR1 on AtGPA1 is not shown, and the combination of AtGPA1 and GCR1 null mutations indicated the existence of a G-protein-independent function for GCR1 in brassinosteroid (BR) and GA responses [73]. The interaction between GCR1 and AtGPA1 is controversial [59,74].

Using the weak sequence similarity between cAR1 and GCR1 to claim GPCR homology may not be warranted because it is not confirmed whether *D. discoideum* cAR1 has guanine nucleotide exchange activity on Gα protein(s) [75]. In fact, cAR-like proteins are found in organisms lacking G proteins. Furthermore, the human genome encodes around 800 GPCR genes, but none are homologous to *D. discoideum*'s cAR1 gene [76], and no homologous gene encoding a non-cAR GPCR is found in plant genomes [76]. Therefore, without homology support or biochemical proof and without the need for a GEF to activate a plant Gα-subunit, there is no compelling reason to designate GCR1 as a GPCR. Among other plant GPCR candidates published, GCR2 was disproved because it lacks a 7TM domain [77,78] and its published ABA-related phenotypes [68] are not reproducible [78–80]. MLO proteins are the only plant 7TM candidates to have a proved GPCR topology; however, there is no genetic evidence that MLOs regulate G proteins [81].

Recently, GTG1 and GTG2 were proposed to be plant GPCRs. GTG proteins are highly homologous to the human GPR89a that was erroneously annotated as GPCR 89 [60]. The human GPR89a protein is a voltage-dependent anion channel and is now re-annotated as a Golgi pH transporter [61]. This biochemical function for GTG proteins is supported by a recent study [82] showing that GTG1 is localized primarily in Golgi bodies and in the endoplasmic reticulum, but not in the plasma membrane, raising doubts about whether GTG proteins function as GPCRs. It should be emphasized that not all seven transmembrane proteins function as GPCRs. Instead, they also have other functions such as ion channels in insects, channel rhodopsins in green algae or bacterial rhodopsins [83–85]. Finally, many of the so-called plant GPCRs have homologues in organisms that lack G signalling altogether, indicating that the constrained evolutionary function is something other than G protein activation [86]. Therefore, plant biologists must exercise extreme caution in interpreting plant GPCR functionality, especially because some human GPCR-like genes, such as those called orphan receptors, are not yet proved to have G-protein-dependent functions [87].

## 4. G protein components and their regulators in plants

The *Arabidopsis* genome contains one canonical Gα (AtGPA1) [7], one Gβ (AGB1) [30] and three Gγ (AGG1, AGG2 and AGG3) genes [31,32,88]. This is roughly the G protein inventory for most diploid plants; for example, rice encodes one canonical Gα, one Gβ and 5 Gγ-subunits [89]. The few species having more than one Gα-subunit are recent polyploids, and there is no reason to conclude that these Gα-subunits evolved subfunctions [12]. Loss-of-function mutants of AtGPA1 and AGB1 share phenotypes, including altered sugar sensing [21,22,90,91], stomate closure [13,38] and seedling development [26,37], whereas *agb1* mutants, but not *gpa1* mutants, show increased lateral root production [37] and are hypersensitive to fungal infection (table 1) [16,18,39]. *agg1* and *agg2* mutants singly and in combination selectively phenocopy the AGB1 null mutant in pathogen resistance, development and sugar sensing [39,93], whereas the triple *agg1 agg2 agg3* mutant displays all the AGB1 null mutant phenotypes examined [88,94]. Therefore, as in mammalian systems, the Gβγ dimer functions as one signalling element and not as free Gβ- or Gγ-subunits.

**Table 1.** Characteristic morphological phenotypes of heterotrimeric G protein subunit mutants in *Arabidopsis* and rice. n.d., not determined.

mutant	seedling (etiolated)	seedling (light-grown)	mature plant	references
*Arabidopsis*				
Gα (*gpa1*)	short hypocotyl, open apical hook	fewer lateral root	round leaves, reduced root mass, long sepals, wide silique	[26,27,37]
Gβ (*agb1*)	short (shorter than *gpa1*) hypocotyl, open apical hook	more lateral root	round leaves, small rosette size, increased root mass, short sepals, short and wide silique with blunt tip, reduced height	[27,36,37,39,88]
Gα Gβ (*gpa1 agb1*)	phenocopy *agb1*	phenocopy *agb1*	phenocopy *agb1*	[27]
Gγ1 (*agg1*)	wild-type-like	more lateral roots	wild-type-like	[39]
Gγ2 (*agg2*)	wild-type-like	more lateral roots	wild-type-like	[39]
Gγ3 (*agg3*)	short hypocotyl	more lateral roots	phenocopy *agb1* except reduction in rosette size and small differences in flower and silique size	[88,92]
Gγ1Gγ2 (*agg1 agg2*)	wild-type-like	more lateral roots	wild-type-like	[93]
Gγ1Gγ2Gγ3 (*agg1 agg2 agg3*)	short hypocotyl	phenocopy *agb1*	phenocopy *agb1*	[94]
rice				
Gα (*rga1*, *d1*)	n.d.	dwarf	dwarf, erected leaf, short panicle, short seed	[28,29,95,96]
Gβ (*rgb1RNAi*)	n.d.	dwarf	dwarf, reduced size of panicles, browning of the lamina joint regions and nodes, reduced seed size (short length and width), reduced seed number and fertility	[97]
*rgb1RNAi/d1–5*	n.d.	dwarf	similar to *rgb1RNAi* with more severe phenotypes	[97]
Gγ (*gs3*)	n.d.	n.d.	a major quantitative trait locus (QTL) for grain length and weight, and a minor QTL for grain width and thickness	[98,99]
Gγ (*dep1*)	n.d.	n.d.	gain-of-function mutation results in a reduced length of the inflorescence internode, an increased number of grains per panicle and an increase in grain yield	[100]

G protein γ-subunits exhibit an extraordinary level of structural diversity (figure 5) and show important differences to their animal counterparts [89]. While all animal γ-subunits are very small proteins (less than 100 amino acids), AGG3 is 251 amino acids long and some AGG3 homologues can be in excess of four times the average mammalian size (the rice AGG3 homologue DEP1 is 426 amino acids long). Another important difference is that many plant γ-subunits do not contain an isoprenylation motif at their C-terminus, an obligate requisite in all animal γ-subunits and essential for membrane anchoring. At this time, there are three classes of γ-subunits based on their structure [89]. Type A groups the prototypical γ-subunits, small in size and containing a C-terminal CaaX isoprenylation motif (CaaX means cytosine, then any 2 aliphatic residues and then, X, any residue). Type B γ-subunits are similar to type A, still small in size but lacking the CaaX motif, or indeed any cysteine residues at the C-terminal end of the protein. Type C γ-subunits have two well-defined regions: an N-terminal domain with high similarity to classic γ-subunits and a C-terminal domain highly enriched in cysteine residues [89]. Importantly, *Arabidopsis* does not have a type B γ-subunit, with AGG1 and AGG2 both being type A subunits, whereas AGG3 is type C. *Arabidopsis* AGG1 and AGG2 and rice Gγ1 (RGG1) have the prototypical Gγ architecture [27–29]. Rice Gγ2 (RGG2) lacks the prenylation site [29]. *Arabidopsis* AGG3 has an N-terminal Gγ domain, a weakly predicted transmembrane helix near the centre, and a C-terminal-cysteine-rich region [72]. The rice genome encodes three AGG3 homologues: GS3, DEP1 and G protein γ-subunit type C 2 (OsGGC2) [73]. Compared with the Gγ-domain possessing typical length and predicted secondary structure, the cysteine-rich domain is highly divergent among different species [73].

In addition to the single canonical Gα-subunit, *Arabidopsis* has three extra-large G protein genes (*XLG1*, *XLG2* and *XLG3*), composed of a C-terminal Gα-like domain and an N-terminal extension containing a nuclear localization signal and a cysteine-rich region (figure 5) [57]. The XLG proteins bind and hydrolyse guanine nucleotides [101], interact with Gβ [102], localize primarily to the nucleus [103] and exhibit physiological functions in root morphogenesis [103]. While typical α-subunits use small bivalent cations (e.g. magnesium, calcium or manganese) for optimal nucleotide binding and hydrolysis, XLG proteins require an extremely low amount of calcium as cofactor, but not magnesium. These findings are not consistent with structural predictions [58]; thus our understanding of these unusual Gα-subunits is unclear, and future work will undoubtedly yield new surprises.

**Figure 6.**
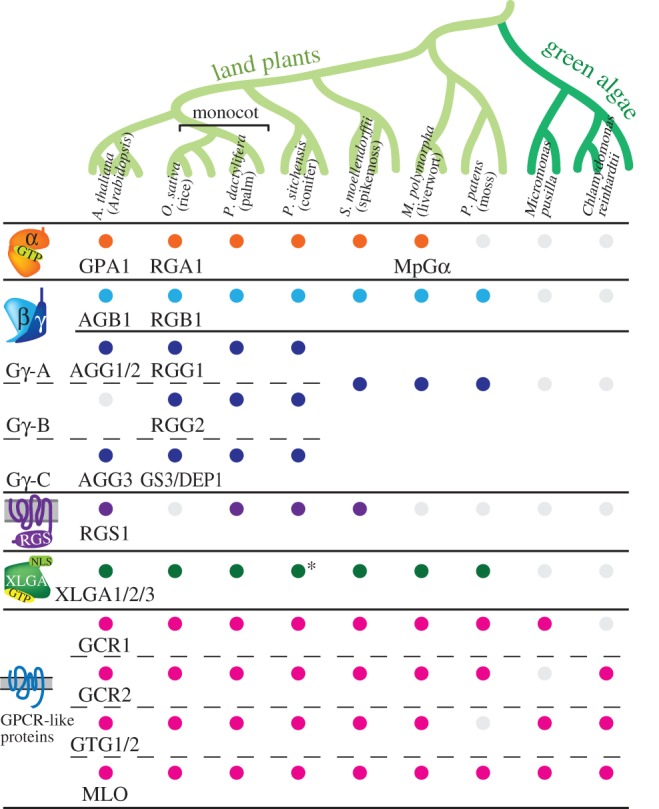
G protein components in the plant kingdom. Homologous genes of *Arabidopsis* G protein components (the α-, β- and γ-subunits), AtRGS1, XLG and 7TM proteins were searched using the blast program. The candidates of homologues were further evaluated by the membrane topology, domain structure, and other featured sequences. Coloured dots show conservation of homologous genes. See also [48,89] for the phylogenetic trees, accession numbers and Gγ classes. Asterisk denotes a conifer XLG which is not registered in NCBI (nr/nr) data for *P. sitchensis*, but is found in EST data for *Picea glauca*.

Figure 6 summarizes the presence of G protein components and regulators in representative species in eudicots (*Arabidopsis thaliana*), monocots (*Oryza sativa* and *Phoenix dactylifera*), gymnosperms (*Picea sitchensis*), spikemosses (*Selaginella moellendorffii*), bryophytes (*Marchantia polymorpha* and *Physcomitrella patens*) and green algae (*Micromonas pusilla* and *Chlamydomonas reinhardtii*) [48]. With few exceptions, the G protein components (α-, β- and γ-subunits) and the RGS genes are either found or deleted together in each genome [104]. This suggests that these four proteins function together, and deletion of one of the components from the genome releases the other elements from an evolutionary constraint to keep them intact in the genome. Homologous genes of *Arabidopsis* Gα, Gβ, Gγ and XLG are broadly found in land plants, except the moss, *P. patens*, which encodes no Gα homologous gene [48]. No G protein elements are yet found in the sequenced green algae genomes. The 7TM-RGS genes are found in eudicots, a monocot (date palm), gymnosperms and *Selaginella*, but not in the fully sequenced genomes of true grasses (e.g. rice and maize), bryophytes and green algae [48]. Both the 7TM domain and the RGS box of the 7TM-RGS genes are well conserved throughout the land plants, suggesting that the two domains were fused early during plant evolution [48].

The two ‘RGS-less’ exceptions in plants raise the opportunity to find still another mechanism for G protein activation. While monocots have 7TM-RGS proteins, the cereals, a subgroup of monocots, and the liverwort *M. polymorpha* lack functional RGS proteins [48]. Interestingly, the liverwort Gα-subunit hydrolyses its GTP rapidly in the absence of any regulatory protein (*M. polymorpha* MpGα, *k*_cat_ = 0.87 min^−1^) [48] likely to compensate for the loss of the 7TM-RGS protein in liverwort. This drastic difference in the intrinsic property of the liverwort Gα-subunit indicates that an intrinsic regulatory feature of signalling molecules is constrained or determined by the binding partner, and that a loss of the regulator gene may lead to a drastic change of intrinsic property of the target molecule during evolution. In contrast to liverwort, the rice G protein is self-activating—like AtGPA1, because of its rapid nucleotide exchange (RGA1; *k*_on_ = 0.9–2.4 min^−1^) and slow hydrolysis of GTP (RGA1, *k*_cat_ = 0.05 min^−1^) [48,105]. No regulatory element of rice G protein has been identified so far, but the intrinsic property of RGA1 implies that it is regulated by unknown GAP proteins. These observations make study of rice and liverwort signalling of foremost importance; no doubt we will find even more bizarre mechanisms controlling the G protein activation state.

It should be noted that GCR1, GCR2, GTG and MLO proteins are well conserved in land plants and green algae, even in species lacking encoded Gα and/or the other G protein components in their genomes (figure 6). The homologies between the *A. thaliana* and the green alga *M. pusilla* genes are supported by high expectation values (AtGCR1, *E*-value = 7*e*^−21^; AtGTG1 *E*-value = 1*e*^−37^; and AtMLO1, *E*-value = 2*e*^−95^). Normally, genes losing their functional partners are released from genetic constraint, quickly mutate and become deleted from the genome (neutral theory of evolution [106]). The lineage of green algae was separated from land plants more than 1 billion years ago [107]. Therefore, these GPCR-like genes conserved in green algae probably have a function irrelevant to G signalling [86].

## 5. The G protein effectors and the plant G protein interactome

Table 2 lists plant G protein interactors that are partially characterized. Animal G proteins have adenylyl cyclases and other well-known effectors; however, plant genomes do not encode the canonical G protein effectors [121]. Among many G protein interactors [91,115,116,119–123], some function as potential G protein effectors, such as thylakoid formation 1 (THF1) for Gα [91] and ACI-reductone dioxygenase 1 for Gβγ [119], although effectors identified so far are not sufficient to explain divergent functions of plant G proteins. An international consortium of plant G protein researchers undertook a focused screen to identify, *ab initio,* plant G protein effectors [42]. The interactors include many proteins having different intracellular localizations, physiological functions and domain architectures. Some of them are completely uncharacterized proteins, but others have well-defined domains, such as kinases, phosphatases and transcription factors. Within the set of kinases, AtWNK8 phosphorylates AtRGS1 and induces AtRGS1 endocytosis [53]. Interestingly, AtRGS1 is predicted to interact with some receptor-like kinases (RLKs) [42]. The *Arabidopsis* genome has more than 600 genes in the RLK gene family, and many of them have known ligands and signal transduction pathways. Because there are genetic links between the RLKs, such as ERECTA [16,36], and heterotrimeric G proteins, it is possible that the RLKs may transmit signals to G proteins through the phosphorylation and endocytosis of AtRGS1. As mentioned earlier, despite the small subset of the genes, plant G proteins operate in many signal pathways; adding the RLK to the mix is a possible explanation of how so many signals can propagate through this G protein nexus. Because plant G proteins are detected in a huge protein complex *in vivo* [33,124], the RLKs or other unidentified proteins would compose the stable machinery with G proteins.

**Table 2.** List of partially characterized heterotrimeric G protein interactors in *Arabidopsis*.

protein	encoded protein	relation to G-proteins	function	references
physical interactors				
AtRGS1	a predicted seven transmembrane protein with C-terminal RGS box	preferentially bind GTP-bound AtGPA1 and exhibit GTPase-accelerating protein activity on AtGPA1	attenuate cell elongation in hypocotyls; attenuate cell division in primary roots; upregulate the expression of ABA biosynthesis genes; positively regulate d-glucose sensing in high concentrations of d-glucose-inhibited hypocotyl elongation, root elongation and cotyledon greening	[23,27,40,51,108,109]
XLG1	has a C-terminal Gα-like domain and an N-terminal extension containing a nuclear localization site and a cysteine-rich region		negatively regulate primary root growth; negatively regulate ABA inhibition of seed germination	[57,103]
XLG2	has a C-terminal Gα-like domain and an N-terminal extension containing a nuclear localization site and a cysteine-rich region	co-immunoprecipitated with AGB1	negatively regulate primary root growth; negatively regulate ABA inhibition of seed germination; enhanced susceptibility to *P. syringae*; regulate floral transition	[101–103]
XLG3	has a C-terminal Gα-like domain and an N-terminal extension containing a nuclear localization site and a cysteine-rich region		negatively regulate primary root growth; negatively regulate ABA inhibition of seed germination; positively regulate root waving and root skewing	[103,110]
GCR1	a 7TM protein with weak sequence homology with the cAMP receptor, cAR1, of the slime mould	bind AtGPA1	negatively regulate ABA inhibition of seed germination, early seedling development and gene expression; negatively regulate ABA-inhibited stomate opening and ABA-promoted stomate closure; positively regulate GA- and BR-stimulated seed germination; promote blue light-induced gene expression; promote cell division in tobacco BY-2 suspension cells	[22,59,65,66,73,111–113]
GCR2	a predicted membrane protein with sequence homology with members of the eukaryotic lanthionine synthetase component C-like protein family	contradictory AtGPA1 binding	contradictory ABA binding and ABA receptor role (ABA inhibition of seed germination, early seedling development, and root elongation, and ABA-induced gene expression)	[68,77,79,80,114]
GTG1 GTG2	predicted to consist of eight to 10 transmembrane domains with sequence homology to hamster GPHR, an anion channel critical for Golgi acidification and function	bind AtGPA1; have intrinsic GTP binding and GTPase activity; its GTPase activity is inhibited by AtGPA1	a putative ABA receptor; contradictory role in the regulation of ABA inhibition of seed germination, post-germination growth and ABA-induced gene expression; positively regulate ABA-induced promotion of stomate closure; regulate fertility, hypocotyl and root growth, and responses to light and sugars	[60,82]
AtPIRIN1	a member of the cupin protein superfamily	bind AtGPA1	negatively regulate ABA signalling in seed germination and early seedling development; mediate blue light-induced gene expression	[113,115]
PLDα1	a major isoform of phospholipase D	bind AtGPA1 and exhibit GAP activity on AtGPA1	produce phosphatidic acid; positively regulate ABA-inhibited stomate opening and ABA-promoted stomate closure	[116,117]
THF1	a plastid protein; no significant sequence homology with other proteins	bind AtGPA1	act downstream of AtGPA1 to regulate d-glucose sensing; promote chloroplast development with AtGPA1	[91,118]
PD1	a cytosolic prephenate dehydratase	bind AtGPA1	regulate blue light-mediated synthesis of phenylpyruvate and phenylalanine and gene expression	[67,113]
ARD1	ACI-reductone dioxygenase 1	bind AGB1 and AGB1-AGG1	overexpression of ARD1 suppresses the 2-day-old etiolated phenotype of *agb1–2*; AGB1 stimulates the enzymatic activity of ARD1.	[119]
NDL1 NDL2 NDL3	N-MYC downregulated-like, a predicted members of a lipase superfamily containing an NDR domain and an *α*/*β* hydrolase fold.	bind AGB1-AGG1 and AGB1-AGG2	a positive modulator of primary root growth and lateral root formation; positively modulate basipetal and negatively modulate acropetal auxin transport in an AGB1-dependent manner; work together with AGB1 to regulate primary root length and lateral root density through modulation of auxin transport; AGB1 is required for NDL1 protein stability in regions of the root where auxin gradients are established	[120]

There are a few findings that should be noted but still do not make sense. Wang's group reported that AtGPA1 directly inhibits activity of the phospholipase, PLDα [105], and that this enzyme possesses a ‘DRY’ motif [116] found on GPCRs that is important for G activation in animals. The PLDα interaction is to the GDP form and purportedly stimulates steady-state hydrolysis, but this is unexpected because increased hydrolysis dictates interaction with the GTP-bound form or the transition state. Moreover, their published PLDα ‘DRY’ sequence was shown to be incorrect [125], and there are no follow-up publications of this exciting finding, suggesting this avenue may be a dead end. Similarly, a claim is made that pea and wheat Gα-subunit interacts with PLC [11,126], but, again, with no follow-up in 5 years and the fact that plant PLCs do not have the G activation ‘bells and whistles’ of the corresponding animal PLC enzymes, there is cause for pause and bewilderment.

## 6. Physiological function of G proteins

**Figure 7.**
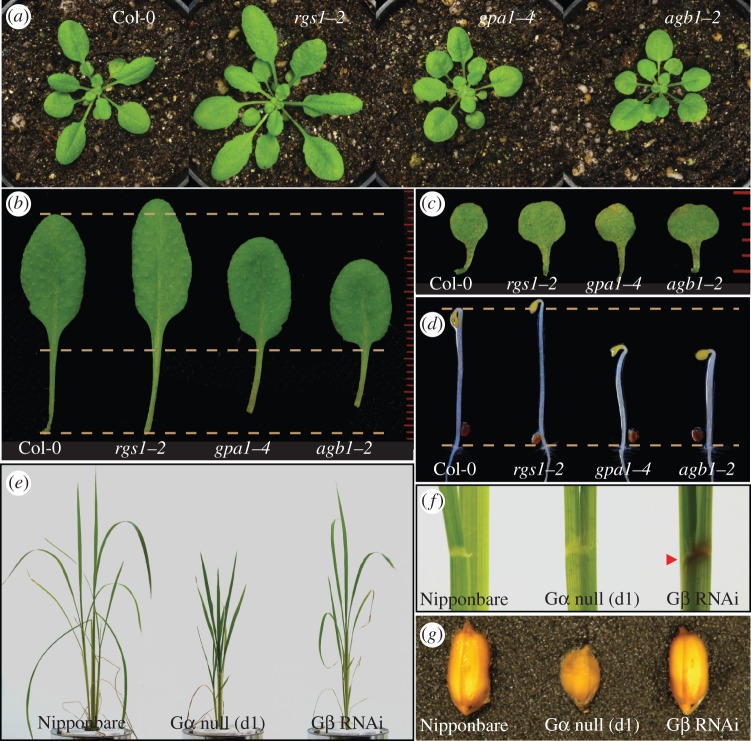
Morphology of loss of G protein mutants in *Arabidopsis* and rice. (*a–c*) Growth and leaf shape; *Arabidopsis* T-DNA insertion lines for Gα (*gpa1–4*), Gβ (*agb1–2*) or RGS1 (*rgs1–2*) and wild-type Col-0 were grown for 37 days in a short day chamber (8 L : 16 D cycle, 100 μmol m^−2^ s^−1^) at 23°C. Cotyledons (*c*) or ninth leaves (*b*) are shown with a scale. (*d*) Two-day-old etiolated seedlings; *Arabidopsis* T-DNA lines were grown vertically on half of MS plate containing 1% d-glucose under dark condition at 23°C. (*e*) Growth of rice; Nipponbare (wild-type), the Gα knockout (d1, DK22) or Gβ knockdown (5-4-1) lines were grown in a short day chamber (8 L : 16 D cycle, 34 C during day per 28 C during night time, 320 μmol m^−2^ s^−1^) for 47 days. (*f*) Colour of joint region; lamina joint regions of fourth leaves of Nipponbare and the G protein mutants. Gβ knockdown line shows brown colour [97]. (*g*) Seed shape; rice seeds for wild-type Nipponbare, Gα knockout (DK22) or Gβ knockdown (5-4-1) lines. Gα knockout causes abnormal round shape of seeds [28,29].

This section summarizes physiological functions found from loss-of-function G protein mutants in *Arabidopsis* and rice (figure 7; tables 1 and 3).

**Table 3.** Response of heterotrimeric G protein subunit mutants to plant hormones and glucose in *Arabidopsis* and rice. Note that this list does not include disease and transcriptional responses. n.d., not determined.

mutant	auxin	ABA	GA	BR	other hormones	glucose	references
*Arabidopsis*							
Gα (*gpa1*)	wild-type-like response to auxin inhibition of hypocotyl and primary root elongation, reduced sensitivity to auxin in lateral and adventitious root formation	increased sensitivity to ABA in seed germination and early seedling development, and inhibition of primary root elongation; hyposensitive to ABA inhibition of stomatal opening and ABA-inhibition of the inward K^+^-channels	reduced sensitivity to GA in seed germination	reduced sensitivity to BR in seed germination, hypocotyl and root elongation	wild-type response to ACC-induced triple response and ACC promotion of seed germination	hypersensitive to high concentration of glucose inhibition of seed germination, early seedling development and root growth	[13,21,22,37,38,73,91,127]
Gβ (*agb1*)	wild-type-like response to auxin inhibition of hypocotyl and primary root elongation, increased sensitivity to auxin in lateral and adventitious root formation	increased sensitivity to ABA inhibition of seed germination, early seedling development and primary root elongation and lateral root formation; hyposensitive to ABA in ABA inhibition of stomatal opening and ABA inhibition of the inward K^+^-channels	reduced sensitivity to GA in seed germination	reduced sensitivity to BR in seed germination	hyposensitive to methyl jasmonate inhibition of root elongation and seed (paclobutrazol-pre-treated) germination	hypersensitive to high concentration of glucose inhibition of seed germination, early seedling development and root growth	[18,21,22,38,73,127]
Gα Gβ (*gpa1 agb1*)	near wild-type response to auxin in lateral root formation (without NPA pre-treatment)	hypersensitive to ABA inhibition of seed germination, early seedling development, primary root elongation and lateral root formation; hyposensitive to ABA inhibition of stomatal opening and ABA-inhibition of the inward K^+^-channels	n.d.	n.d.	n.d.	increased sensitivity to glucose-induced inhibition of seed germination	[22,38]
Gγ1 (*agg1*)	increased sensitivity to NAA in lateral root formation in NPA-pre-treated seedlings; negatively modulates acropetal auxin polar transport in roots	wild-type responses to ABA in seed germination and stomatal movement	n.d.	n.d.	n.d.	hypersensitive to high concentrations of d-glucose (and mannitol) inhibition of seed germination	[39,93,127]
Gγ2 (*agg2*)	increased sensitivity to NAA in lateral root formation in NPA-pre-treated seedlings; negatively modulates basipetal auxin polar transport in roots	wild-type responses to ABA in seed germination and stomatal movement	n.d.	n.d.	n.d.	hypersensitive to high concentrations of d-glucose (but not mannitol) inhibition of seed germination	[39,93]
Gγ3 (*agg3*)	n.d.	hypersensitive to ABA inhibition of seed germination, stomatal opening and the inward K^+^-channels	n.d.	n.d.	n.d.	hypersensitive to 2% sucrose rescue in ABA inhibition of seed germination assay	[88]
Gγ1Gγ2 (*agg1 agg2*)	increased sensitivity to NAA in lateral root formation in NPA-pre-treated seedlings; negatively modulates basipetal auxin polar transport in roots	wild-type responses to ABA in seed germination and stomatal movement	n.d.	n.d.	n.d.	hypersensitive to high concentrations of d-glucose (and mannitol) inhibition of seed germination	[39,93]
Gγ1Gγ2Gγ3 (*agg1 agg2 agg3*)	n.d.	n.d.	n.d.	n.d.	n.d.	n.d.	[94]
rice							
Gα (*rga1*, *d1*)	n.d.	n.d.	hyposensitive to GA-promoted α-amylase induction and seed germination	hyposensitive to 24-epi-BR inhibition of root growth, the inclination of leaf lamina, the promotion of coleoptile and second leaf sheath elongation	n.d.	n.d.	[95,128,129]

### 6.1. Growth and morphology

The analysis of loss-of-function alleles and transgenic lines of *Arabidopsis* Gα (*AtGPA1*) and Gβ (*AGB1*) makes it clear that G proteins mediate processes throughout development. Knockout mutants of AtGPA1 and AGB1 display and share developmental phenotypes, from seed germination to flower and silique development [21,26,36,37] (figure 7*a*–*e*), except that *agb1*, but not *gpa1*, promotes lateral root production [37]. Although loss-of-function alleles of *Arabidopsis* Gγ1 (*AGG1*) and Gγ2 (*AGG2*) appear to be largely normal, *agg3* single and *agg1 agg2 agg3* triple mutants exhibit many similar morphological phenotypes previously observed in *agb1* mutants [39,88,93,94], indicating that the repertoire of Gγ-subunits in *Arabidopsis* is complete [94]. Table 1 lists visible morphological phenotypes reported in G protein mutants. Four of these phenotypes are frequently described as characteristic of G protein mutants: (i) short hypocotyl and open apical hook in etiolated seedlings in *gpa1* and *agb1* mutants; (ii) round-shaped rosette leaves in *gpa1*, *agb1*, *agg3* and *agg1 agg2 agg3* mutants; (iii) reduced lateral root formation in *gpa1* mutant and increased lateral root formation in *agb1* mutants; and (iv) *erecta*-like flower morphology in *agb1* and *agg1 agg2 agg3* mutants.

Although the precise cause of these growth and morphological phenotypes of G protein mutants is unclear, many of them are attributed to their fundamental cellular defects in cell division or elongation. For example, the short hypocotyl in *gpa1* and *agb1* etiolated seedlings is due to reduced axial cell division in hypocotyl epidermal cells [26,37]. The round-shaped rosette leaves in *gpa1* mutants contain larger and fewer epidermal cells, presumably owing to increased cell expansion compensating reduction in cell division [26]. The reduced lateral root formation in *gpa1* mutant and increased lateral root formation in *agb1* mutant is largely due to altered activity in lateral root primordia, again pointing to a modulatory role on cell proliferation [37]. However, the molecular mechanism underlying the regulation of cell proliferation by G protein remains unclear. Because overexpression of AtGPA1 in synchronized tobacco BY-2 cells shortens the G1 phase of the cell cycle and promotes the formation of nascent cell plate [26], AtGPA1 may modulate the cell cycle at the G1-to-S transition phase.

Some striking differences in growth and morphological phenotypes are observed between *Arabidopsis* and rice G protein mutants (table 1 and figure 7). The dwarf rice *d1* mutant has dark green and broad leaves as well as compact panicles and short grains [130]. While *Arabidopsis* G protein mutants, *gpa1* and *agb1*, are largely similar to wild-type plant in terms of height, both rice G protein mutants, *rga1* and *rgb1*, are dwarf [28,29,95,97]. Moreover, two rice type C Gγ-subunits, *grain size 3* (*GS3*) and *dense and erect panicle 1* (*DEP1*), are important quantitative trait loci for grain size and yield [89,98–100], and mutations in both rice Gγ-subunits enhanced yield. In *Arabidopsis*, *gpa1*, *agb1* and *agg3* mutant seeds are shorter and wider [88], contrasting to the seed phenotype of the *gs3* mutant. These findings suggest that cereals may use G protein signalling mechanisms distinct from other flowering plants. For example, a key regulator of G protein signalling (RGS) protein has been discovered in *Arabidopsis* but not in rice. A single amino substitution found in grass Gα is responsible for the physical decoupling of the RGS protein and its cognate Gα partner [48]. This may help explain some fundamental differences in growth and morphological phenotypes observed in G protein mutants between *Arabidopsis* and rice.

### 6.2. Hormone and glucose responses

Plant hormones regulate every aspect of plant growth and development. Given that G protein mutants display an array of phenotypes, it is not surprising that G protein mutants show alterations in responses to multiple plant hormones. Table 3 lists all published responses of G protein mutants to plant hormones.

The most direct and compelling evidence for the involvement of G protein in a plant hormone response came from the work on rice dwarf mutant, *d1* [28,29,95]. *d1* was initially identified as a GA-insensitive mutant. Map-based cloning revealed that the defect in the *d1* mutant was, in fact, due to a loss-of-function mutation in the gene encoding Gα, *RGA1*. Consistent with a role in GA, signalling, *d1* rice aleurone cells are markedly less sensitive to GA, as quantitated by transcription of α*-amylase* and *OsGAMYB* that encodes a GA-inducible transcription factor that positively regulates the expression of α*-amylase*. Similar to the rice *d1* mutant, *Arabidopsis* G protein mutants also display reduced sensitivity to GA in seed germination [21,73].

*gpa1*, *agb1* and *agg3* mutants all are hypersensitive to ABA inhibition of seed germination [21,22,88], early seedling development and root elongation [21,22], and ABA-induced gene expression [22]. On the other hand, these mutants are hyposensitive to ABA inhibition of stomatal opening and inward K^+^-channels [13,38,88]. These findings suggest that G proteins function as both negative and positive regulators of ABA signalling, depending on the specific cell type. Consistent with a role of G proteins in ABA signalling, several AtGPA1-interacting proteins regulate ABA responses. For example, similar to *gpa1* mutants, the *gcr1* mutants are hypersensitive to ABA inhibition of seed germination, early seedling development and ABA-induced gene expression [22]. Similarly, *pirin1* mutants are hyposensitive to ABA inhibition of seed germination and early seedling development [115].

G proteins are not essential for auxin responses because G protein mutants have wild-type responses to auxin inhibition of hypocotyl growth and primary root elongation [37]. However, G protein mutants have altered sensitivity to auxin in lateral root formation. While *gpa1* mutants are less sensitive to auxin, *agb1*, *agg1*, *agg2* and *agg1 agg2* mutants are more sensitive to auxin in lateral root formation [37,88,127]. Data from follow-up studies of these observations indicate that G proteins modulate auxin transport. The effect of the auxin polar transport inhibitor, *N*-1-naphthylphthalamic acid (NPA), either applied at the shoot–root junction (to block polar auxin transport from the shoot) or at the root tip (to block basipetal auxin polar transport from the root tip), proves that AGG1 (together with AGB1) acts within the central cylinder to attenuate signalling from acropetally transported auxin, and that AGG2 (together with AGB1) affects the action of basipetally transported auxin within the epidermis and/or cortex [127]. These findings indicate that the Gγ-subunits provide functional selectivity in Gβγ dimer signalling.

Arguably the most known about the role of G proteins is in sugar signalling. All G protein subunit mutants are hypersensitive to glucose in seed germination [21,22,88,91,127]. *gpa1* and *agb1* are hypersensitive to glucose inhibition of early seedling development and root elongation [21,22,91]. The loss-of-function of AtRGS1 confers glucose hyposensitivity [23,40,51,73]. Glucose also attenuates G protein modulation of lateral root formation [90]. Because glucose regulates AtRGS1 activity towards AtGPA1 [40], and glucose stimulates the endocytosis of AtRGS1 protein and subsequently physically uncouples the GAP activity of AtRGS1 from AtGPA1, permitting the sustained activation of AtGPA1 [53], it has been proposed that AtRGS1 functions as a receptor or co-receptor for glucose. Considering the regulatory role of glucose in diverse hormone biosynthesis and signalling, it is possible that many of the hormone sensitivity phenotypes observed in G protein mutants may be due to altered glucose signalling. An attractive hypothesis is that plant hormone signalling integrates information about the plant nutrient status. This information is relayed, in part, by the RGS1 pathway.

Glucose and auxin merged recently in research on G-protein-mediated root growth and development [90,120,131,132]. Root architecture is established and maintained by gradients of auxin and nutrients such as sugars. Auxin is transported acropetally through the root within the central stele and then, upon reaching the root apex, auxin is transported basipetally through the outer cortical and epidermal cells. In *Arabidopsis*, AGG1 and AGG2 are differentially localized to the central and cortical tissues of the root, respectively. AGB1/AGG dimers bind a protein designated N-MYC downregulated-like1 (NDL1) [120]. NDL proteins act in a signalling pathway that modulates root auxin transport and auxin gradients in part by affecting the levels of at least two auxin transport facilitators. Gain- and loss-of-function mutations place NDL proteins central to root architecture through a direct effect on auxin transport and auxin maxima patterns. Feedback controls involving AGB1, auxin and sugars are required for NDL1 protein stability in the regions of the root where auxin gradients are established.

Finally, *gpa1* and *agb1* mutants are hyposensitive to BR [21,26,128,129,133] in BR promotion of seed germination [26,73], and in BR inhibition of hypocotyl and root elongation [26]. *agb1* mutants are hyposensitive to methyl jasmonate (MeJA) inhibition of root elongation and seed germination [18]. Rice *rga1* mutants are hyposensitive to 24-epi-BR inhibition of root growth, the inclination of leaf lamina, the promotion of coleoptile and second leaf sheath elongation [128,129].

In addition to hormonal and sugar responses, G proteins are involved in light responses [24,134,135], in mechanical sensing at the root tip [130] and in calcium response to extracellular nucleotides [136], although the detailed mechanisms are unknown.

### 6.3. Stomatal movements and ion channel regulation

Stomata allow plants to exchange gases and water with the atmosphere. Oxygen and carbon dioxide diffuse out of or into leaves for photosynthesis, and water is lost from the leaves through transpiration. Stomatal opening and closing are regulated by environmental signals (e.g. light and humidity), plant hormones and pathogen infection [137–139]. The stomatal aperture size is determined by a pair of guard cells, which change cell shape through turgor pressure. The cell turgor is determined by ionic strength, gated by transmembrane flux of K^+^, Cl^−^ and malate [138,139]. In addition, cytosolic calcium and reactive oxygen species (ROS) function as second messengers to regulate the ionic strength and stomatal movements [138,139].

G proteins are involved in ABA-induced stomatal movements by controlling inward- and outward-rectifying potassium current or an anion channel [13,14,38,88,140]. Loss-of-function mutants of *Arabidopsis* AtGPA1, AGB1 or AGG3 are impaired in ABA-dependent inhibition of inward K^+^ channels, and are hypersensitive to ABA in inhibiting light-induced stomatal opening [13,38,88]. In *agb1*, *agg3* or *gpa1 agb1* mutant leaves, stomatal movement does not occur either with ABA or light, and K^+^ flux is not changed with ABA treatment [38,88]. However, the ABA-dependent regulation is not impaired in the Gγ1/Gγ2 double null plants [93], suggesting that the Gβγ3 (AGB1/AGG3) complex specifically regulates this pathway. The Gα null plant has wild-type responsiveness to the pathogen bacterial peptide flg22 known to inhibit inward K^+^ channels and stomatal opening [140].

In addition to ion channel regulation, G protein mutations affect water availability [141,142], but this cannot be explained simply by aberrant stomate development [143]. *gpa1* null mutations confer reduced, but *agb1* or *rgs1* mutations confer increased stomate density on cotyledons [142]. The *gpa1* mutations also confer reduced stomate formation on mature leaves [141].

*Arabidopsis* G protein mutants have altered responsiveness to ozone [20], a chemical that elicits a bimodal oxidative burst in leaf cells [19,144]. Both *gpa1* and *agb1* mutants lack the early peak in the ozone-induced oxidative burst, whereas only *gpa1* lacks the second peak [19]. In addition, when exposed to ozone, *gpa1* mutants are more sensitive to damage, whereas *agb1* mutants are less sensitive than wild-type plants. G proteins are also involved in the production of cytoplasmic H_2_O_2_ necessary for the stomata closure induced by extracellular calmodulin (ExtCaM), also dependent on nitric oxide accumulation [15,62]. ExtCaM induces an increase in H_2_O_2_ levels and cytosolic calcium, leading to a reduction in stomatal aperture. *gpa1* mutants are impaired in ExtCaM-induced production of H_2_O_2_ in guard cells and the subsequent stomata closure. ExtCaM-mediated NO generation is regulated by AtGPA1, whereas AtGPA1 activation of NO production depends on H_2_O_2_. Finally, the involvement of G proteins is not confined to the induction of ROS; *agb1* mutants are more sensitive to H_2_O_2_ than wild-type plants, suggesting that G proteins also influence sensitivity to ROS [63].

### 6.4. Pathogen resistance

In order to confront a huge variety of pathogens, plants use a two-tiered defence strategy; the primary defence recognizes conserved microbial molecules called pathogen-associated molecular patterns (PAMPs; sometimes referred to as microbe-associated molecular patterns) and trigger a response known as PAMP-triggered immunity (PTI). The second tier recognizes specific pathogen-effector proteins, unleashing the effector-triggered immunity (ETI). The PTI response is elicited by a variety of membrane-bound pattern recognition receptors that recognize PAMPs such as flagellin from bacterial pathogens or chitin from fungal pathogens. During evolution, some pathogens developed strategies to overcome PTI using virulence effectors, but plants acquired ETI.

The involvement of G proteins in plant defence was suspected early on [64,145]. The first evidence was obtained using chemical modulators of G protein activity, although these chemicals have questioned specificity. Cultured soya bean cells treated with an antigen-binding antibody fragment recognizing a highly conserved fragment in the Gα-subunit show a ten-fold enhancement of the elicitor-induced oxidative burst, whereas heat-inactivated antibody has no effect [146]. In addition, the synthetic peptide mastoparan, an activator for inhibitory Gα-subunits in animals, cause a typical defence-associated oxidative burst even in the absence of elicitors, although it should be noted that the action of mastoparan in plants has been called into question [147]. Transgenic tobacco plants expressing cholera toxin (CTX) under the light-inducible promoter have reduced susceptibility to *Pseudomonas tabaci*, accumulate high levels of salicylic acid and display constitutive expression of several pathogenesis-related (PR) genes [148]. CTX covalently modifies stimulatory Gα-subunits in animals, but its mode of action in plants is unknown. G proteins and the oxidative burst seem to be linked by activation of phospholipase C (PLC); however, if true, this would be by a mechanism dissimilar to animal systems [149] because plant PLCs lack domains known for G protein activation [150]. In potato, treatment with a non-hydrolysable analogue of GTP, which results in G protein activation, inhibited resistance to *Phytophthora infestans* [151]. Soya bean suspension cultures pre-treated with suramin, a G-protein inhibitor, lack the typical oxidative burst induced by *Pseudomonas syringae* pv. *glycinea* harbouring the avrA (avirulence) gene [152]. Additional direct and indirect evidence using chemical modulators links plant G proteins to defence responses [153–161], although chemical treatments could produce artefacts owing to uneven tissue penetration and the documented lack of specificity in plants [147,162].

The *Arabidopsis* and rice mutants prove the involvement of G proteins in defence. Rice *d1* mutant alleles are susceptible to an avirulent race of the fungal pathogen *Magnaporthe grisea*, the causal agent of the rice blast disease [163]. Induction of the PR genes *PR1* and *PBZ1* compared with wild-type plants are delayed in the *d1* mutant. Several *d1* mutants treated with *M. grisea* sphingolipid elicitors produce little H_2_O_2_ and fail to induce the *PBZ1* gene [163]. Interestingly, by day 2 the steady-state level of *RGA1* mRNA decreases upon infection with a virulent race of *M. grisea* and increases by an avirulent race, especially at the points of infection [163], indicating that induction of *RGA1* expression is R-gene-dependent. In rice, the production of defence-related ROS is mediated by a small GTPase, OsRac1. OsRac1 acts downstream of RGA1 in the production of H_2_O_2_ in response to *M. grisea* elicitors, but not in the expression of PR genes, emphasizing the complexity of the mechanism. The RGA1/OsRac1 defence pathway uses the mitogen-activated protein kinases (MAPK). OsMAPK6 is post-translationally activated by an *M. grisea* sphingolipid elicitor, and silencing of the gene severely suppresses the elicitor-activated expression of the PR protein, phenyl ammonia-lyase [164]. Both *d1* and *OsRac1* mutants strongly reduce the elicitor-induced OsMAPK6 activation as well as the OsMAPK6 protein levels, indicating that OsMAPK6 acts downstream of both G proteins. The link between OsMAPK6 and OsRac1 is further substantiated by co-immunoprecipitation experiments showing that OsMAPK6 interacts with the active OsRac1 but not with the inactive form. The lignin biosynthetic enzyme OsCCR1 and the ROS scavenger metallothionein (OsMT2b) are also regulated by OsRac1, and could be involved in the RGA1/OsRac1 defence pathway, although a direct link with RGA1 has not yet been demonstrated [165,166].

In contrast to the rice Gα-subunit, *Arabidopsis* Gα's involvement in defence is limited, and in fact *Arabidopsis gpa1* mutants have slightly increased resistance to several pathogens. The link between G proteins and plant defence in *Arabidopsis* is nevertheless clearly established through the Gβγ dimer [16,18]. Mutants deficient in AGB1 are more susceptible to the fungal pathogens *Alternaria brassicicola*, *Botrytis cinerea*, *Fusarium oxysporum* and *Plectosphaerella cucumerina* [16,18]. Upon infection with *A. brassicicola* or treatment with methyl jasmonate MeJA, *agb1* mutants show a significant delay in the induction of the MeJA-induced PR genes PDF1.2, OPR3 and PAD3 [18], whereas expression of the salicylic-acid-dependent PR1 was increased after infection with *P. cucumerina* [16]. The above-mentioned fungal pathogens are necrotrophs (or in some cases considered hemi-biotrophs, i.e. undergoing biotrophic and necrotrophic phases during their life cycle). When *agb1* mutants are challenged with the bacterium *P. syringae* and the oomycete *Peronospora parasitica*, they do not show differences compared with the wild-type plants [16,18]. Cell wall callose deposition, a typical response to pathogen attack, is greatly reduced in *agb1* mutants challenged with *P. cucumerina*, but not with *P. parasitica*. The increased susceptibility to necrotrophic fungi and the delayed induction of the MeJA-related PR genes suggests an involvement of AGB1 in the MeJA-mediated defence pathway. This hypothesis is supported by the decreased sensitivity displayed by the *agb1* mutants to several MeJA-induced developmental phenotypes [18].

Three different Gγ-subunits potentially confer functional selectivity to the Gβγ dimer [39]. To investigate such selectivity, the involvement of all three Gγs in defence was studied, with AGG1 being clearly implicated in the response against *F. oxysporum* and *A. brassicicola* [39]. *agg1* mutants are hypersensitive to *F. oxysporum* and *A. brassicicola* [39], the role of AGG2 is unclear, and AGG3 has no defence-related role [88]. Transgenic plants expressing AGG2 under the control of the AGG1 promoter complement *agg1* mutants and restore resistance to wild-type levels, indicating that the defence specificity observed for AGG1 does not reside in its primary sequence and is transcriptional or post-transcriptional (L. Thung & J. S. Botella 2013, unpublished results) [167]. *agb1* mutants are hypersensitive to the pathogen *P. cucumerina,* whereas *gpa1*, *agg1* and *agg2* mutants display similar levels of sensitivity to wild-type plants [168]. The double *agg1 agg2* mutant exhibits identical sensitivity levels to *agb1*, implicating both Gγ-subunits in the defence against this pathogen. The level of resistance in all mutants is correlated with lower xylose content in the cell wall [42,168].

Aside from the canonical subunits, one of three extra-large α-subunit XLGs [57], XLG2, is linked to plant defence [102]. *xlg2* mutants have enhanced susceptibility to *P. synringae* and reduced induction of the pathogenesis-related gene *PR2*. Microarray analysis revealed that, aside from *PR2*, other pathogen-inducible genes are downregulated in *xlg2* mutants in response to *P. syringae* infection, whereas overexpression of XLG2 resulted in the production and accumulation of aberrant transcripts for several defence-related genes [102]. XLG2 physically interacts with AGB1, but the interaction is restricted to infected tissues. Interestingly, in contrast to *agb1* mutants, *xlg2* mutants show wild-type levels of resistance to the necrotrophic pathogens *B. cinerea* or *A. brassicicola* [102].

In addition to the direct resistance evidence obtained for a number of pathogens, G proteins are associated with plant defence responses such as cell death and the oxidative burst. Rice *d1* mutants reduce hypersensitive response (HR)-associated cell death upon infection with avirulent races of *M. grisea* [163]. AtGPA1, but not AGB1, is required for the cell death observed in response to ozone treatment in *Arabidopsis* [19]. *agb1* mutations decrease cell death induced by tunicamycin, an antibiotic that inhibits N-linked protein glycosylation, implicating AGB1 in the unfolded protein response (UPR) [169,170]. The UPR is activated in response to disruption of the protein folding machinery and results in apoptotic cell death in mammalian systems [171]. Although the UPR is not well characterized in plants, it is well established that the endoplasmic reticulum's secretory machinery is important in plant immunity [169]. G proteins are also involved in phytochrome A-mediated cell death that occurs when hypocotyls of far red-grown seedlings are exposed to white light [63]. In contrast to the UPR, in this case, *agb1* mutants show increased cell death, whereas *gpa1* mutants show decreased cell death.

A rapid increase in ROS is observed following recognition of a pathogen by plants. Although ROS are intimately linked to the plant immune response, they also play important signalling roles in development, hormonal response and abiotic stress [19,144,172,173]. Pathogen-induced ROS production in rice contrasts with that in *Arabidopsis* G protein mutants. Rice *d1* mutant cell cultures treated with elicitors derived from *M. grisea* show a reduced H_2_O_2_ production, perhaps explaining the reduced resistance shown by the mutant [163]. Or perhaps not, because even though *agb1* mutants are hypersensitive to *P. cucumerina*, no reduction in ROS production is observed upon infection with the pathogen [16,18].

These profound differences in pathogen resistance in rice and *Arabidopsis* G mutants bring us back again to one of the ‘take home’ lessons from this review. Because of mechanistic differences in G activation, it is important that both rice and *Arabidopsis* be adopted as models for G signalling research. For example, it is clear that G proteins play very different roles in *Arabidopsis* and rice defence, possibly owing to the absence of RGS proteins in grasses that might have resulted in divergent functional evolution at least for the α-subunit. Therefore, G protein mutants need to be produced and studied in other species before a ‘universal’ picture can be revealed.

## 7. Summary and perspective

Phenotypes of the loss- and gain-of-function mutants of G protein components, their regulators and the proposed effectors leave no doubt that plant G signalling does not follow in step with animal G signalling. The vast knowledge from the field of animal science is therefore of limited value to researchers studying G protein activation mechanisms in plants. This global difference is largely due to the unique ‘self-activating’ property of plant G proteins (see §3). The identification of G protein effectors and regulators will definitely advance the field. Great progress was made recently through a genome-wide screening for physical interactions with key signalling components in the G protein pathway in *Arabidopsis* [42]. It behoves the plant biology community to take advantage of this valuable plant resource (http://bioinfolab.unl.edu/emlab/Gsignal/index.pl).

As for regulatory molecules, several 7TM proteins were identified as GPCR candidates, but there is no proof of their GEF activities. The rate-limiting step of the plant G protein cycle is different from that of animals. It appears that in most plants, if not all, the GTP hydrolysis is the rate-limiting step, and 7TM-RGS modulates the hydrolysis rate. However, G protein regulators are absent in the grasses, where 7TM-RGS genes cannot be found in fully sequenced genomes, implying that other proteins possessing GAP activity may modulate GTP hydrolysis in grasses. Furthermore, plant G proteins are apparently involved in divergent physiological processes, but the mechanism of how the G protein system perceives the extracellular stimuli remains unclear. We proposed that endocytosis of AtRGS1 by a kinase pathway decouples the ‘self-activating’ G protein from the negative regulator. Therefore, it will be informative to determine whether other potential ligands for G protein pathways (e.g. ABA and other hormones), in addition to d-glucose and other sugars, promote the AtRGS1 phosphorylation and endocytosis. Also, because several receptor kinases, including ERECTA [16,36], are genetically related to G protein mutants, it is interesting to test if the kinases directly phosphorylate AtRGS1 and promote its endocytosis. When the regulator candidates are identified, a biochemical approach is preferable to clarify the functionality *in vitro*. In parallel, use of FRET to measure *in vivo* activation of the Gα–Gβγ complex is needed [124]. The downstream G protein effectors are also unclear, although much progress has been made in this arena (table 2).

In conclusion, *Arabidopsis* and rice have emerged as important model systems to advance our understanding of G signalling beyond what we have learned using animal cell lines and fungi. Because plants are the most distant eukaryotes from opisthokonts (e.g. animals and fungi) and have distinct G protein systems, plants make it possible to address the evolution of G signalling and network architecture. However, much is still to be done; a complete set of effectors and a better understanding of the apical reactions in G signalling are sorely needed.

Because G signalling is at the heart of many plant physiologies of agronomic importance, such as disease resistance and harvest index, translational work on G signalling will certainly improve agriculture [174]. The last 10 years brought great surprises, and we predict more to come in the next 10 years.

## 8. Acknowledgements

We thank Dr Yukimoto Iwasaki for sharing rice Gα- and Gβ-mutant seeds, and Dr Kimitsune Ishizaki for searching *M. polymorpha* genes. Work in the Jones Laboratry is supported by grants to A.M.J. from the NIGMS (R01GM065989), NSF (MCB-0723515 and MCB-1158054), and the Genomic Science Program, US Department of Energy, Office of Science, Biological and Environmental Research (DE-FG02-05ER15671). J.-G.C.'s research is supported by the Plant–Microbe Interfaces Scientific Focus Area in the Genomic Science Program, US Department of Energy, Office of Science, Biological and Environmental Research. Oak Ridge National Laboratory is managed by UT-Battelle, LLC, for the US Department of Energy under contract no. DE-AC05-00OR22725. Work in J. Botella's laboratory is supported by the Australian Research Council (DP1094152).
